# Clinical and microbiological effects of adjunctive photodynamic diode laser therapy in the treatment of chronic periodontitis: A randomized clinical trial

**DOI:** 10.34172/joddd.2020.030

**Published:** 2020-09-21

**Authors:** Sahana Mallineni, Sreenivas Nagarakanti, Sumanth Gunupati, Ramesh Reddy BV, Mahaboob V Shaik, Vijay K Chava

**Affiliations:** ^1^Department of Periodontology, Narayana Dental College & Hospital, Nellore, India; ^2^Department of Genetics, Advanced Research Centre, Narayana Medical College & Hospital, Nellore, India

**Keywords:** Chronic periodontitis, Photodynamic therapy, Hotosensitizing agents, Polymerase chain reaction, Porphyromonas gingivalis

## Abstract

**Background.** Conventional mechanical debridement alone cannot eliminate bacteria and their products from periodontal pockets. Adjunctive therapies improve tissue healing through detoxification and bactericidal effects. Photodynamic therapy (PDT) is a non-invasive treatment procedure that involves the use of a dye as a photosensitizer to attach to the target cell and be activated by a photon of an appropriate wavelength. This study aimed to assess the effectiveness of PDT in treating periodontitis as an adjunct to scaling and root planing.

**Methods.** Fifteen subjects with chronic periodontitis were treated randomly with scaling and root planing (SRP), followed by a single PDT (test) or SRP (control) episode alone. Full-mouth plaque index (PI), sulcus bleeding index (SBI), probing depth (PD), and clinical attachment level (CAL) were assessed at baseline and 1-month and 3-month intervals. Microbiological evaluation of Porphyromonas gingivalis (Pg) in subgingival plaque samples was performed using a commercially available real-time polymerase chain reaction.

**Results.** The results revealed a significant difference in PI, SBI, PD, CAL, and microbiological parameters between the groups one and three months after treatment.

**Conclusion.** A combination of PDT and SRP gave rise to a significant improvement in clinical and microbiological parameters in patients with chronic periodontitis.

## Introduction

Periodontitis is considered the most common oral disease in response to the chronic infection caused by different periodontopathogenic bacteria, resulting from inflammation of structures supporting teeth.^1^

It has been shown that conventional scaling and root planing (SRP) result in significant clinical improvements, but they do not completely remove periodontopathogens, especially in deep periodontal pockets^2,3^ and cannot prevent bacterial invasion into periodontal soft tissues.^4^ SRP might even favor bacteremic and endotoxemic events.^5,6^

Another crucial issue in the treatment of periodontitis is that periodontopathogenic bacteria can penetrate and persist in epithelial cells of the periodontal pockets and superficial gingiva,^7,8^ thus evading host immunity and conventional antimicrobial drugs. This might predispose to the recolonization of periodontal tissues after treatment and disease relapse.^9^

In this era of the scientific explosion, there is increasing awareness about microbial resistance-related phenomena.^10^ Resistance development might be the consequence of the excessive use of antibiotics in general bacterial or viral infections.

Although systemic and local antibiotics have been used as an adjunct to conventional therapy, the outcome of periodontal therapy is questionable due to its unfavorable side effects and drug resistance.

To overcome these limitations and deliver better results, a novel mode of non-invasive and effective therapy has been developed, referred to as photodynamic therapy (PDT). This approach has emerged in recent years as a non-invasive treatment modality for many infections caused by bacteria, viruses, and fungi.^11^ It is also used in oral cancer care and the photodynamic diagnosis of malignant oral lesion transformation.^12^

PDT is a modality of medical care that uses light with different wavelengths to activate a photosensitizing or photoactive agent (photosensitizing agent) in the presence of oxygen.^11^

Periodontal diseases are mainly bacterial infections that live in plaque biofilms. On average, the significant differences in subgingival plaque composition between periodontal health and disease are the higher total bacterial counts and the increased counts of red complex species. Red complex species increase significantly in numbers with increased pocket depths.

Porphyromonas gingivalis (Pg), a red complex organism, is a significant human periodontal pathogen. Detection of different bacterial species in subgingival biofilm samples might help determine an individual’s disease risk, the essence of optimal periodontal therapy, and microbial post-therapy outcomes. The prevalence and quantity of Pg in subgingival plaque samples are determined by anaerobic culture and the real-time polymerase chain reaction (RT-PCR). It was possible to detect as few as one colony-forming Pg unit by the RT-PCR assay.^13^

This study compared the clinical and microbiological outcomes of SRP with or without the adjunctive use of a single episode of PDT in patients with chronic periodontitis, testing the hypothesis that the adjunctive use of PDT might improve the outcomes of non-surgical periodontal care.

## Methods

A clinical, split-mouth, triple-masked, randomized, controlled trial was performed in compliance with the revised Helsinki Declaration of 1975 in 2013.

Study Design and Patient Selection

The protocol for this study was confirmed by the Institutional Ethics Board, Nellore, Andhra Pradesh, India (NDC/PG/Diss/2015-16/EC/2015). The trial was registered with the Clinical Trials Registry, India (CTRI/2017/09/009634). Fifteen patients presenting with untreated chronic periodontitis were recruited from the Department of Periodontology. A minimum sample size of 15 patients was estimated to detect a 50% difference in intergroup comparisons with a power of 80% at the P=0.05 level of significance.

The patients were aware of the study procedures and signed informed consent forms from December 2016 to July 2017 to participate in the trial for three months. The study’s inclusion criteria of the study were as follows: previously untreated chronic periodontitis, with at least twenty permanent natural teeth with at least one permanent premolar and molar in each quadrant with a probing depth of ≥5 mm in each quadrant. Criteria for exclusion consisted of systemic disorders and conditions affecting the outcomes of periodontal therapy, intake of systemic antibiotics or pharmaceuticals (possibly influencing periodontal condition) within the last six months, using tobacco in any form, pregnant and lactating women, participants with poor oral hygiene, and those refusing to adhere to the protocol.

### Procedural steps

After a detailed case history report that included the chief complaint, clinical review, and thorough periodontal examination, clinical and microbiological parameters were recorded at different time intervals. Plaque index (PI),^14^ sulcus bleeding index (SBI),^15^ probing depth (PI), and clinical attachment level (CAL)/relative attachment level (RAL) were recorded at baseline (before SRP), and one and three months after treatment. Microbiological parameters were evaluated at baseline and after three months postoperatively by collecting subgingival plaque samples. The plaque samples were obtained by inserting a Gracey curette subgingivally into the deepest portion of the periodontal pocket parallel to the tooth’s long axis, and by gently scraping along the root surface coronally. A blind investigator not engaged in the treatment process reported all the parameters at different stages. The microbial samples were stored in Tris-EDTA medium and sent for RT-PCR.

### Microbiological PCR analysis

The microbial plaque samples were stored in Tris-EDTA medium with patients’ IDs at -20ºC.

### DNA isolation

Deoxyribonucleic acid (DNA) isolation was carried out by kit method (cat.no.MB505), applying the instructions of the HiPurATM Bacterial Genomic DNA Purification Kit (HiMedia Laboratories Pvt. Ltd. Mumbai, 400 086, India).

### Primers

RT-PCR detection of putative periodontal pathogen Pg in subgingival specimens was carried out by the 16S ribosomal RNA gene. Two Pg-specific primers described by Slots et al^16^ were used to amplify a 404-bp fragment of the 16S rRNA gene

P. gingivalis 16S rRNA gene forward (Primer 1):( 5’-AGG CAG CTT GCC ATA CTG CG-3’)

P. gingivalis 16S rRNA gene reverse (Primer 2):(5’-ACT GTT AGC AAC TAC CGA TGT-3’)

To obtain a standard curve, P. gingivalis primers and P. gingivalis DNA positive control (Bioserve Biotechnologies Pvt. Ltd. Hyderabad, Luna® Universal qPCR Master Mix [cat. No. NEB #M3003S-New England Biolabs, Inc. Ipswich, MA 01938-2723, USA]) were used.

### Quantitative PCR

Dye-based quantitative PCR (qPCR) was used to quantify DNA amplification as it occurred during each cycle of a PCR in real-time fluorescence of a double-stranded DNA (dsDNA) binding dye (SYBR® GREEN I). A quantification period, or Cq value, was calculated at a point where the fluorescence signal was confidently detected over the background fluorescence.

Cq values were used to determine relative target abundance between two or more samples or to measure absolute target quantities based on an acceptable standard curve obtained from a set of known dilutions.

Luna Universal qPCR Master Mix and other reaction components were thawed at room temperature and placed on ice. After thawing completely, each component was briefly mixed by inversion, pipetting, and gentle vortexing. “SYBR GREEN” channel of the real-time instrument BIORAD-CFX100 (BIORAD, USA) was used to quantify P. gingivalis, using Luna Universal Master Mix. Amplification reactions were performed in a total volume of 25 μL, consisting of 10 µL of 1X Luna Universal qPCR Master Mix (which contains deoxynucleoside triphosphate (dATP, dTTP, dCTP, and dGTP; MgCl2, Taq buffer, SYBR Green, polymerase), 1 µL of Pg forward primer (10 µM), 1 µL of Pg reverse primer (10 µM), 10 µL of template DNA (P. gingivalis DNA standard/plaque DNA sample) and 3 µL of Nuclease-free Water.

All the mix was prepared in 96-well hard-shell PCR plates (WHT-CLR, cat.no.HSP 9601) (Bio-Rad Laboratories Inc, US) with seal plates of optically transparent film (Bio-Rad Laboratories Inc., US).

Care was taken to properly seal the plate edges and corners to prevent artifacts caused by evaporation. PCR amplification was performed in a real-time thermocycler (BIORAD-CFX100, BIORAD, USA).

Cycling conditions were as follows: initial denaturation at 95°C for one minute; 40 cycles consisting of 95°C for 30 s, 60°C for one minute and 72°C for one minute; and a final extension at 72°C for two minutes. For copy number determination to make a standard curve in the PCR set-up, the positive control, P. gingivalis DNA template was prepared at five different concentrations by serial dilutions: 1×109, 1×107, 1×105, 1×103, and 1×101 copy numbers. This was used to generate a standard curve of P. gingivalis copy number/CT value. For the negative control, 10 µL of the RNAse/DNAse-free water was added instead of the template DNA.

Treatment Procedure

All the patients received periodontal care by the same clinician, which included full-mouth SRP. Additionally, one quadrant (the test group) was handled with PDT using a split-mouth design. The control and test groups were allocated based on a table of random numbers generated by a computer. The list was obscured before the administeration of interventions.

For the test group, after SRP, the periodontal pockets of the selected teeth were filled with toluidine blue (PAD Plus viscous solution, manufactured by Denfotex research. Ltd), from the bottom of the pocket using a blunt needle. After three minutes of dwell time, the photosensitizer was rinsed with a saline solution to remove excess photosensitizer. The pocket was then exposed to the diode laser (Siro Laser Xtend –Dentsply), using 635-nm laser beams, at 0.8-W energy, with a 300-μm fiber optic tip for 60 seconds.

The participants were instructed in proper brushing technique, and the clinical parameters were recorded at one and three months, postoperatively. The collected data were subjected to statistical analysis, and the results were presented under the headings of various parameters considered for this study.

### Statistical analysis

Data analysis was carried out using SPSS 22 (IBM SPSS, Armonk, NY, IBM Corp). Shapiro–Wilk test showed normal distribution of all the clinical parameters. Therefore, parametric methods were applied for data analysis.

## Results

With unpaired t-test, the study and control groups’ clinical parameters were evaluated at different time intervals. For both groups, comparisons were made using paired t-test. Differences at P<0.05 were deemed statistically significant.

PI showed a mean difference of 0.20±0.19 and 0.40±0.63 from the baseline to one month and three months, respectively, in all the participants, which was significant (P=0.001) (Table 1).

**Table 1:** Comparison of plaque index in all the patients at different time intervals

**Clinical parameters**		**Difference (Mean ± SD)**	**t-value**	**P-value**
**Baseline**	**One month**	0.24±0.18	5.06	<0.001*
	**Three months**	0.20±0.19	4.03	0.001*

Paired t-test: P<0.05 (*statistically significant), P>0.05 (Not significant-NS)

SBI exhibited no significant difference (P=0.208) between the control (1.86±0.35) and test (1.66±0.48) groups at baseline. Both groups had lower SBI values at one and three months after treatment than the baseline, with significantly lower test group values than the control group (Table 2).

**Table 2:** Comparison of clinical and microbiological parameters between the control and test groups at different time intervals

**Clinical parameters**	**Test group** **(Mean ± SD)**	**Control group** **(Mean ± SD)**	**t-value**	**P-value**
**Sulcus bleeding index**
**Baseline**	1.66±0.48	1.86±0.35	1.288	0.208
**One month**	0.80±0.56	1.40±0.50	3.074	0.005*
**Three months**	0.66±0.48	1.26±0.59	3.024	0.005*
**Probing depth**
**Baseline**	5.93±0.79	5.66±0.72	0.958	0.346
**One month**	4.06±0.88	5.13±0.83	3.400	0.002*
**Three months**	3.73±0.88	4.93±0.88	3.556	0.001*
**Clinical attachment level**
**Baseline**	7.20±0.94	5.86±1.50	0.645	0.007*
**One month**	4.33±1.11	5.40±1.54	1.434	0.039*
**Three months**	3.60±0.98	5.33±1.54	2.751	0.010*
**PCR**
**Baseline**	1.76±1.54	1.50±1.11	0.519	0.608
**One month**	0.83±1.02	1.80±0.84	2.800	0.009*
**Three months**	0.53±0.82	1.57±1.36	2.512	0.018*

Unpaired t-test: P<0.05 (*statistically significant), P>0.05 (Not significant-NS)

Baseline PDs were not significantly different between the control and study groups. In both groups, PDs at one month and three months after treatment showed a substantial decrease, with a higher effect in the study group than the control group (Table 2).

There was a substantial difference in CAL postoperatively at one month and three months intervals in both groups, with a significant improvement in the study group, compared to the control group (Table 2).

Baseline PCR values exhibited no significant difference between the two groups. There was no significant difference between the one-month and three-month values after treatment in the control group; however, there was a statistically significant difference in the test group (Tables 2 and 3).

**Table 3:** Intra group comparison of microbiological parameters in the control and test groups at different time intervals

		**(Mean ± SD)**	**t-value**	**P-value**
**PCR (control group)**
**Baseline**	**One month**	0.29±1.44	0.79	0.440
	**Three months**	0.06±1.70	0.15	0.879
**PCR (test group)**
**Baseline**	**One month**	0.92±1.46	2.44	0.028*
	**Three months**	1.22±1.42	3.33	0.005*

Paired t-test: P<0.05 (*statistically significant), P>0.05 (Not significant-NS)

## Discussion

PDT was first used as a tool for cancer care in the medical field over a century ago.^17^ Several studies have shown the efficacy of PDT in periodontal care with specific photosensitizing dyes.^1,18^

A split-mouth randomized clinical trial was performed to determine the effectiveness of PDT in treating chronic periodontitis as an adjunct to SRP. On the basis of a computer-generated random number table, 30 sites in 15 patients were randomly selected and randomly assigned to the control and test groups.

The present study showed that, in line with previous research, the clinical and microbiological outcomes of non-surgical periodontal care (SRP) of chronic periodontitis improved by the adjunctive use of PDT.^19,20^

PDT is regulated by singlet oxygen, which affects the extracellular molecules directly. Thus, the polysaccharides present in a bacterial biofilm’s extracellular matrix of polymers are also susceptible to photodamage;^21^ such dual activity is displayed and can represent a significant advantage of PDT.

In the present study, the use of a split-mouth configuration was justified, as the photosensitizer alone cannot produce an antimicrobial effect without laser activation, because only the test site was irradiated.^1,22^ There is a significant reduction in PI values from baseline to 1 and 3 months after treatment in both groups. This can be attributed to a reduction in supragingival plaque after SRP and oral hygiene instructions received during preliminary visits.^23^

There were significant differences in all the clinical parameters (SBI, PD, CAL) in the control and test groups from baseline to one and three months after treatment, which might be attributed to the removal of local etiological factors that harbor multiple pathogenic strains.^23^ There was also a high impact in the study group compared to the control, which might be attributed to the beneficial effect of low-level laser therapy in facilitating wound healing. Positive effects on wound healing following low-level laser therapy can also be due to improved collagen synthesis, reduced inflammation, and increased resistance to wound traction.^1,18,24^

After a three-month evaluation time, there was a significant reduction in SBI in the test group compared to the control group. Reductions in SBI scores in the test group suggest that the combination of photothermal therapy and SRP results in better resolution of inflammation as compared to SRP alone. These results are comparable to previous studies by Raut et al^25^ and Monzavi et al,^26^ where PDT and SRP resulted in a significantly more significant reduction in bleeding scores compared to SRP alone. Several other studies have also concluded that PDT and SRP have an added advantage over SRP alone in the reduction of BoP, thus providing evidence that the combination of PDT and SRP results in better resolution of inflammation.^18,27^

A significant reduction was also noted in PD in the test group compared to the control group. These results are consistent with previous studies where a significant reduction was noted in PD after PDT + SRP.^18,25,27,28^ However, some studies have concluded that PDT and SRP do not result in PD reduction and have no added advantage over SRP.^29,30^ These variations in the results are difficult to interpret because of heterogeneity in study designs and a variety of photosensitizers uses with different laser wavelengths.

There was a significant gain in CAL in the test group in the present study compared to the control group. This gain in the attachment must be due to a decrease in PD as there was a clinically irrelevant gingival recession. Although there was a decrease in PD and gain in CAL, we cannot comment on the attachment type. However, it is most likely to be due to long epithelium formation apart from other contributing factors, such as the removal of local factors and the resultant reduction in inflammation. Although SRP resulted in significant CAL gain, the mean CAL gain was higher in the PDT + SRP group. The gain in CAL in the present study is consistent with the findings of some other studies.^1,25,27^ However, there is still some controversy over the attachment gain after the application of PDT. This has been concluded in various studies reporting that PDT does not affect the attachment gain.^33^

The microbiologic assay (PCR) showed a non-significant reduction in Pg in the control group from baseline to one and three months after treatment. The reduction in microbial flora could be due to SRP and reinforced oral hygiene measures. This is consistent with a study by Cugini et al.^23^ In the test group, there was a significant reduction in Pg compared to the control group at one month and three months after treatment, which might be attributed, in particular, to highly reactive O_2_ molecules, singlet oxygen, and free radicals that can destroy a wide range of proteins, lipids, and carbohydrates, resulting in cell death. These reactive species have detrimental effects on proteolytic enzymes of Pg. Since Pg proteolytic activity is considered an essential mediator of tissue destruction in periodontitis, there might be significant implications for quantitative changes in the development of the related enzymes.^31^

However, some studies showed that PDT, as an adjunct to SRP, did not provide any significant clinical and microbiological benefits.^32-34^ The literature findings reviewed by Meisel and Kocher^35^ indicated that adjunctive PDT does not minimize human periodontal pocket bacterial colonization compared to ultrasonic treatment alone.

A research performed by Polansky et al^36^ concluded that when combined with periodontal instrumentation, a single application of PDT was unable to produce additional clinical and microbiological benefits.

In systematic reviews and meta-analyses performed by Azarpazhooh et al,^37^ Sgolastraet al,^38^ and Peron et al,^39^ it was concluded that PDT as an independent medium/long-term treatment or an alternative to SRP was not superior to regulate the treatment of SRP. Therefore, regular use of PDT for the clinical management of periodontitis cannot be recommended.

The critical limitation of this analysis was the sample size and short duration. Certain drawbacks are that the study used only one laser therapy sequence.

## Conclusion

Within the limitations of this research, it can be concluded that in terms of clinical and microbiological parameters, SRP, combined with PDT, had a significantly better and sustained effect compared to SRP alone. Further work to support the use of PDT in clinical practice should be undertaken with large sample sizes and long-term follow-ups. Future studies should also concentrate on using new generations of photosensitizers that can permeate the outer membrane of gram-negative bacteria to allow the use of non-cationic photosensitizers and multiple laser therapy episodes.

## Authors’ Contributions

All the authors have contributed to the critical revision of the manuscript and approved the final paper. SM, SN, and MVS were responsible for the concept, design, experiment, and data analysis. SG, BVRR, and VKC were responsible for the literature search, drafting, and proof reading of the study. All the authors have read and agreed to the published version of the manuscript.

## Acknowledgments

We would like to acknowledge the participants for their kind cooperation.

## Funding

The study was self-funded; no grants were obtained.

Competing Interests

The authors deny any conflicts of interest with regard to the authorship and/ or publication of this article.

## Ethics Approval

The protocol of this study was approved by the Institutional Ethics Board of Narayana Dental College and Hospital, Nellore, Andhra Pradesh, INDIA (NDC/PG/Diss/2015-16/EC/2015). The trial was registered with Clinical Trials Registry, India (CTRI/2017/09/009634).
